# Stromal Cell-Derived Factor-1β Mediates Cell Survival through Enhancing Autophagy in Bone Marrow-Derived Mesenchymal Stem Cells

**DOI:** 10.1371/journal.pone.0058207

**Published:** 2013-03-05

**Authors:** Samuel Herberg, Xingming Shi, Maribeth H. Johnson, Mark W. Hamrick, Carlos M. Isales, William D. Hill

**Affiliations:** 1 Charlie Norwood VA Medical Center, Augusta, Georgia, United States of America; 2 Department of Cellular Biology and Anatomy, Georgia Regents University, Augusta, Georgia, United States of America; 3 Department of Biostatistics and Epidemiology, Georgia Regents University, Augusta, Georgia, United States of America; 4 Department of Orthopaedic Surgery, Georgia Regents University, Augusta, Georgia, United States of America; 5 Department of Pathology, Georgia Regents University, Augusta, Georgia, United States of America; 6 Institute of Molecular Medicine and Genetics, Georgia Regents University, Augusta, Georgia, United States of America; 7 Institute of Regenerative and Reparative Medicine, Georgia Regents University, Augusta, Georgia, United States of America; The University of Adelaide, Australia

## Abstract

Bone marrow-derived mesenchymal stem/stromal cells (BMSCs) hold great potential for cell-based therapy, yet the therapeutic efficacy remains uncertain. Transplanted BMSCs often fail to engraft within the bone marrow (BM), in part due to the poor survival of donor cells in response to inflammatory reactions, hypoxia, oxidative stress, or nutrient starvation. Two basic cell processes, apoptosis and autophagy, could potentially be responsible for the impaired survival of transplanted BMSCs. However, the functional relationship between apoptosis and autophagy in BMSC homeostasis is complex and not well understood. The stromal cell-derived factor-1 (SDF-1)/CXC chemokine receptor 4 (CXCR4) signaling axis appears to be critical in maintaining proliferation and survival of BM stem cell populations through improving cell proliferation and survival in response to stress; however, the exact mechanisms remain unclear. We recently described novel genetically engineered Tet-Off-SDF-1β BMSCs, which over-express SDF-1β under tight doxycycline-control, thus providing an ideal model system to investigate the isolated effects of SDF-1β. In this study we tested the hypothesis that SDF-1β can mediate cell survival of BMSCs *in vitro* through increasing autophagy. We found that SDF-1β had no effect on BMSC proliferation; however, SDF-1β significantly protected genetically engineered BMSCs from H_2_O_2_-induced cell death through increasing autophagy and decreasing caspase-3-dependent apoptosis. Taken together, we provide novel evidence that the SDF-1/CXCR4 axis, specifically activated by the SDF-1β isoform, plays a critical role in regulating BMSC survival under oxidative stress through increasing autophagy.

## Introduction

Over the last decade, numerous studies have revealed that bone marrow-derived mesenchymal stem/stromal cells (BMSCs) hold great potential for cell-based therapy as BMSCs possess multi-lineage potential [1]. For instance, both autologous and allogeneic BMSCs have been utilized to repair or regenerate bone in experimental and clinical studies [2], [3]. However, attempts to transplant BMSCs from whole bone marrow (BM), enriched peripheral blood, or highly purified low-passage cultures almost universally fail to significantly engraft within the BM when infused into the peripheral circulation of animal and human subjects, in large part due to the poor survival of donor cells [4]–[8]. After being transplanted, BMSCs can face a complex hostile environment with factors that may promote cell loss/death including inflammatory reactions, hypoxia, oxidative stress including reactive oxygen species, and nutrient starvation.

Two basic self-destructive cell processes, apoptosis (‘self-killing’, programmed cell suicide) and autophagy (‘self-eating’, programmed cell recycling), could potentially be responsible for the poor survival and engraftment of transplanted BMSCs used in current cell therapy protocols. Apoptosis, on the one hand, is a set of well-described forms of programmed cell death, which involves the activation of proteolytic enzymes in signaling cascades leading to the rapid destruction of cellular organelles and chromatin [9], [10]. On the other hand, three forms of autophagy have been described, which in general mediate highly regulated mechanisms of cell survival. Macroautophagy (hereafter referred to as autophagy) involves the bulk turnover of cytoplasmic proteins, including damaged or pathologically aggregated proteins, in a generalized fashion as part of a constitutive homeostatic recycling process. Autophagy can be increased in response to stress to provide critically needed nutrients and energy for cellular survival; however, when extreme levels of autophagy are induced, it can also lead to “autophagic cell death” [11]–[13]. Furthermore, autophagy can also specifically target distinct organelles (e.g., mitochondria in mitophagy or the endoplasmic reticulum (ER) in reticulophagy), thereby eliminating supernumerary or damaged cell structures [11], [12]. During autophagy, parts of the cytoplasm and intracellular organelles are sequestered within characteristic double- or multi-membrane autophagosomes and eventually delivered to lysosomes for bulk degradation [11], [12]. Importantly, the functional relationship between apoptosis and autophagy in BMSC homeostasis is complex and not well understood.

Increasing evidence suggests a critical role of the stromal cell-derived factor-1 (SDF-1)/CXC chemokine receptor 4 (CXCR4) signaling axis in maintaining proliferation and survival of BM stem cell populations through improving cell proliferation and survival in response to stress [14]–[16]. It has been speculated that SDF-1 may promote cell survival through two distinct mechanisms: post-translational inactivation of the cell death machinery (e.g., increase anti-apoptotic and decrease pro-apoptotic proteins) and increased transcription of cell survival genes [15], [17]–[20]. In contrast, very little is known about the role of autophagy in stem cells. Three recent reports revealed that MSCs possess high levels of basal autophagy and that further induction of autophagy protects stem cell populations from hypoxia and serum-deprivation-induced cell death [21]–[23]. However, no direct link between the survival-enhancing effects of the SDF-1/CXCR4 axis and autophagy in BMSCs has been established.

Recently, we described genetically engineered Tet-Off-SDF-1β BMSCs that conditionally express SDF-1β; with constitutive SDF-1β overexpression and doxycycline (Dox)-mediated transgene suppression [24]. Constitutive transgene expression results in significantly increased SDF-1β mRNA (30-fold) and protein levels (5-fold) relative to Dox-suppressed and Tet-Off-empty vector (EV) control BMSCs, without altering the normal basal expression of SDF-1 splice variants. Dox control of transgene expression is tightly regulated in a dose-dependent manner with full suppression at 100 ng/ml within 24 hours [24]. Hence, our model, employing both an internal control (+Dox) and a second external empty vector control, allows us to investigate the specific role of SDF-1β in different cell processes with consistent, yet adjustable, cellular expression levels, more akin to continuous endogenous exposure. This avoids potential inconsistencies associated with direct exogenous pre-conditioning, or transient genetic overexpression, where cell exposure levels vary over time or depend largely on the degree of vector transfection. SDF-1β was chosen over the more abundant splice variant SDF-1α due to its greater resistance to proteolytic cleavage suggesting that SDF-1β may be better suited for local applications directly in the BM microenvironment, especially in bone injury sites with increased inflammatory and proteolytic activity [25]–[27]. Given the dearth of scientific literature examining the SDF-1/CXCR4 axis in autophagy and any specific contribution of the SDF-1β isoform to BMSC proliferation and survival, we investigated the role of SDF-1β in H_2_O_2_-induced cell death and tested the hypothesis that SDF-1β mediates cell survival of BMSCs through increasing autophagy, as well as decreasing apoptosis *in vitro.*

## Materials and Methods

### Isolation and Culture of BMSCs

BMSCs were derived from 18-month-old male C57BL/6J mice at the Georgia Regents University Stem Cell Core Facility. Male C57BL/6 mice were purchased from the National Institute on Aging (Bethesda, MD, USA) aged rodent colony. Animals were maintained at the Georgia Regents University - Division of Laboratory Animal Services Facility. All aspects of the animal research were conducted in accordance with the guidelines set by the Georgia Regents University Institutional Animal Care and Use Committee (GRU-IACUC) under a GRU-IACUC approved Animal Use Protocol. The BMSC isolation process, retroviral transduction to express Green Fluorescent Protein (GFP), and clonal selection have been described previously [24], [28], [29]. In brief, six mice were euthanized by CO_2_ overdose followed by thoracotomy. Whole bone marrow aspirates were flushed from femora and tibiae and BMSCs isolated by negative immunodepletion using magnetic microbeads conjugated to anti-mouse CD11b (cat#558013), CD45R/B220 (cat#551513) (BD Biosciences Pharmingen, San Diego, CA, USA), CD11c, and plasmacytoid dendritic cell antigen (PDCA)-1 (cat#130-092-465) (Miltenyi Biotec, Bergisch Gladbach, Germany) followed by positive immunoselection using anti-stem cell antigen (Sca)-1 microbeads (cat#130-092-529) (Miltenyi Biotec) according to the manufacturer’s recommendations. Enriched BMSCs were labeled with GFP [24], [28], [29], and maintained in Dulbecco’s Modified Eagle Medium (cat#10-013) (DMEM; Cellgro, Mediatech, Manassas, VA, USA) supplemented with 10% heat-inactivated fetal bovine serum (cat#S11150) (FBS; Atlanta Biologicals, Lawrenceville, GA, USA). As described in detail, clone 2 was used as the parental cells for further genetic modification with the Tet-Off system at 70–80% confluence [24].

### Genetic Modification of BMSCs for Conditional Expression of SDF-1β

BMSCs were transduced with retroviral Tet-Off expression vectors. The sequential protocol of retrovirus production, two-step infection, and selection to generate double-stable Tet-Off-SDF-1β BMSCs and Tet-Off-EV control BMSCs has been described previously [24]. In brief, 293GPG packaging cells [30] were transfected at passage 8 with retroviral Tet-Off expression vectors containing the SDF-1β coding sequence, or empty control (cat#632105) (Clontech Laboratories, Mountain View, CA, USA). BMSCs (clone 2) were infected at passage 10 with 2 ml of the respective retroviral supernatant containing 4 µg/ml polybrene (cat#H9268) (Sigma-Aldrich, St. Louis, MO, USA) and 100 ng/ml doxycycline (cat#D9891) (Dox; Sigma-Aldrich) followed by selection with 400 µg/ml G418 (cat#091672548) (MP Biomedicals, Solon, OH, USA) and 2.5 µg/ml puromycin (cat#P8833) (Sigma-Aldrich). Clonally selected parental BMSCs and Tet-Off-modified BMSCs were shown to retain their multipotent differentiation potential, including osteogenic potential, over more than 10 passages both *in vitro* and *in vivo* upon transplantation (see [24] and unpublished data). Genetically engineered BMSCs were maintained in DMEM supplemented with 10% Tet-FBS (cat#631106) (Clontech), 400 µg/ml G418, and 2.5 µg/ml puromycin. For *in vitro* experiments, cells at passage 16 were plated at 2.5×10^3^ cells/cm^2^ and then treated with Dox starting the next day. The medium was exchanged daily. To induce cell death, genetically engineered BMSCs were incubated with 1.0 mM H_2_O_2_ or vehicle control for 6 h.

### Cell Proliferation

Cell proliferation of BMSCs in normal growth medium was measured over the course of 7 d using the Vybrant® MTT Cell Proliferation Assay Kit (cat#V13154) (Molecular Probes, Eugene, OR, USA) according to the manufacturer’s recommendation. The assay involves the conversion of the water-soluble MTT (3-(4,5-dimethylthiazol-2-yl)-2,5-diphenyltetrazolium bromide) to an insoluble formazan, which is then solubilized using DMSO and its absorbance measured at 540 nm.

### Cell and Nuclear Morphology

Morphological changes of BMSCs in response to H_2_O_2_ treatment were visualized by phase contrast microscopy. Furthermore, the chromatin dye Hoechst 33342 was used to assess alterations in the nuclear morphology. Cells were washed with PBS, fixed with methanol for 10 min at −20°C, and stained with 5 µg/ml Hoechst 33342 (cat#62249) (Pierce, Thermo Fisher Scientific) for 30 min at room temperature. BMSCs undergoing cell death were visualized by standard phase contrast and fluorescence microscopy using an inverted microscope (Carl Zeiss, Jena, Germany) equipped with an Exfo X-Cite 120 fluorescence lamp (Lumen Dynamics, Mississauga, Ontario, Canada).

### Cell Viability

The viability of BMSCs in response to H_2_O_2_ treatment was analyzed using standard trypan blue exclusion staining. Cells were washed with PBS, lifted with trypsin/EDTA, and resuspended with normal growth medium. Following 1∶5 dilution, BMSC suspensions were mixed with an equal volume of 0.4% trypan blue staining solution (cat#15250061) (Gibco, Invitrogen), and counted in 5 inner squares using a hemacytometer with cover slip (Hausser Scientific, Horsham, PA, USA).

### Western Blotting

Whole cell lysates of BMSCs in response to H_2_O_2_ treatment were prepared in Complete Lysis-M EDTA-free buffer containing protease inhibitors (cat#04719964001) (Roche Diagnostics, Indianapolis, IN, USA). Equal amounts (20 µg) of protein lysates were subjected to SDS-PAGE using 10% NuPAGE® Bis-Tris gels (cat#NP0315BOX) (Invitrogen) and transferred to 0.2 µm PVDF membranes (cat#ISEQ00010) (Millipore, Billerica, MA, USA). Membranes were blocked with 5% non-fat milk in TBST. Apoptosis and autophagy markers were detected using specific primary antibodies (anti-poly(ADP-ribose) polymerase (PARP) (cat#9532), anti-cleaved PARP (cat#9544), anti-cleaved caspase-3 (cat#9664): Cell Signaling Technology, Danvers, MA, USA; anti-beclin 1 (cat#ab16998): Abcam, Cambridge, MA, USA; anti-LC3B-II (cat#R-155-100): Novus Biologicals, Littleton, CO, USA; anti-β-actin (cat#A1978): Sigma-Aldrich, St. Louis, MO, USA) followed by HRP-conjugated secondary antibodies (D-anti-Rb cat#711-035-152; D-anti-Ms cat#715-035-150) (Jackson ImmunoResearch, West Grove, PA, USA). Bound antibodies were visualized with the ECL detection system (cat#34080) (Pierce, Thermo Fisher Scientific) on autoradiography film (cat#E3018) (Denville Scientific, Metuchen, NJ, USA). The intensity of immunoreactive bands was quantified using Photoshop CS4 v11.0 (Adobe Systems, San Jose, CA, USA).

### Statistical Analysis

Experiments were performed three independent times (n = 3–6). All data are expressed as means ± SD. Analysis of variance (ANOVA) followed by Tukey’s or Bonferroni’s *post hoc* test were used to determine mean differences between groups. Null hypotheses were rejected at the 0.05 level. Data were analyzed using GraphPad Prism 5.0 software (GraphPad Software Inc., La Jolla, CA, USA).

## Results

### Cell Proliferation

**Figure 1:**
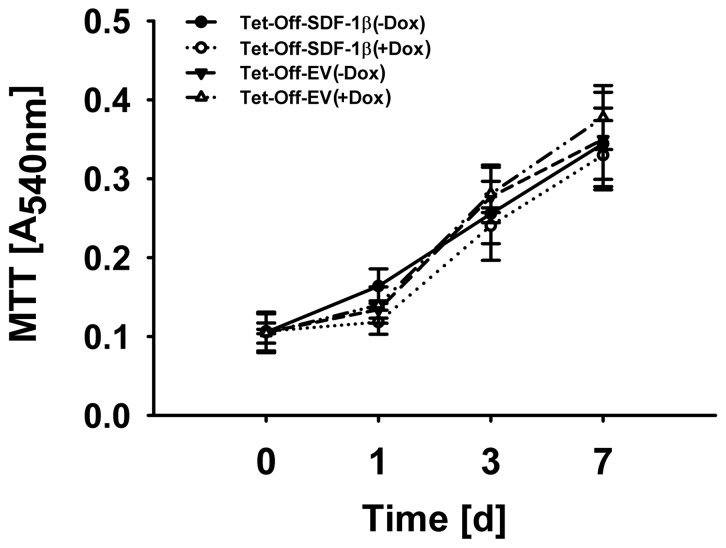
SDF-1β does not affect BMSC proliferation. Colorimetric quantification of DMSO-solubilized MTT formazan at 540 nm showed no differences in proliferation of Tet-Off-SDF-1β compared to Dox-suppressed and Tet-Off-EV controls (1,3, and 7 d, ±100 ng/ml Dox, n = 6, 3 independent experiments).

To investigate the role of SDF-1β in cell proliferation, we cultured BMSCs in normal growth medium and measured the absorbance of DMSO-solubilized MTT formazan at 540 nm after 1, 3, and 7 days (Fig. 1). No differences in cell proliferation were found in Tet-Off-SDF-1β BMSCs compared to Dox-suppressed and Tet-Off-EV controls over the course of 7 d.

### Cell and Nuclear Morphology and Viability

**Figure 2:**
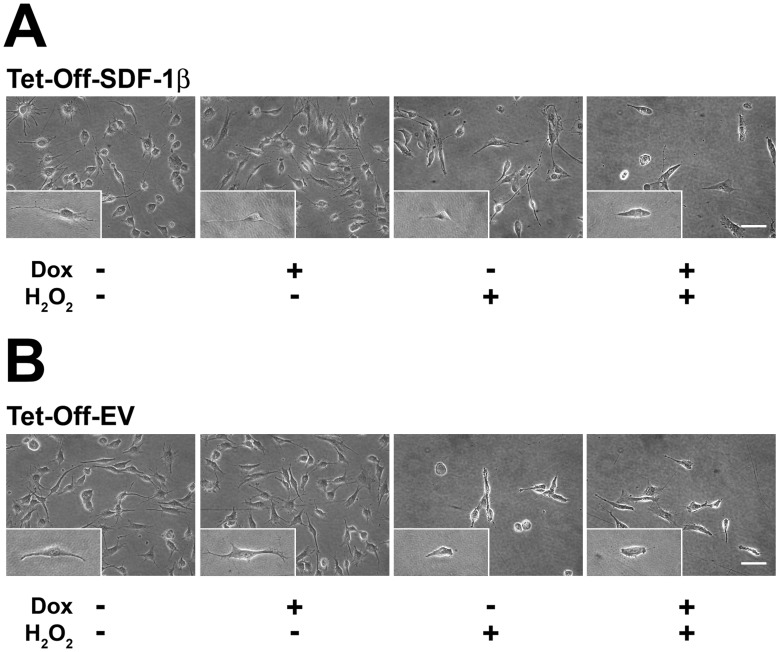
SDF-1β preserves BMSC morphology following exposure to H_2_O_2_. Representative phase contrast micrographs of A) Tet-Off-SDF-1β BMSCs and B) Tet-Off-EV control BMSCs after vehicle control or H_2_O_2_ treatment. Overexpression of SDF-1β in Tet-Off-SDF-1β BMSCs allows for a greater number of cells with preserved morphology after H_2_O_2_ treatment relative to Dox-suppressed and Tet-Off-EV controls (6 h, ±100 ng/ml Dox, ±1.0 mM H_2_O_2_, 20×, 40×, bar 100 µm, n = 3, 3 independent experiments).

**Figure 3:**
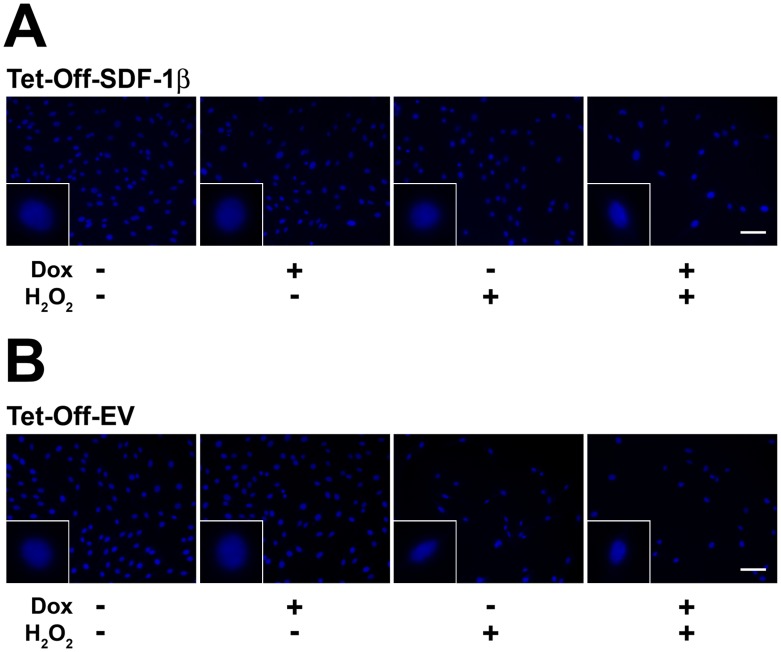
SDF-1β preserves BMSC nuclear morphology following exposure to H_2_O_2_. Representative fluorescence micrographs of Hoechst 33342-stained A) Tet-Off-SDF-1β BMSCs and B) Tet-Off-EV control BMSCs after vehicle control or H_2_O_2_ treatment. Overexpression of SDF-1β in Tet-Off-SDF-1β BMSCs allows for a greater number of surviving cells and cells with preserved nuclear morphology after H_2_O_2_ treatment compared to Dox-suppressed and Tet-Off-EV controls (6 h, ±100 ng/ml Dox, ±1.0 mM H_2_O_2_, 20×, 40×, bar 100 µm, n = 3, 3 independent experiments).

Next, we evaluated the potential role of SDF-1β in protecting BMSCs from cell death. The concentration of H_2_O_2_ necessary to deplete approximately 50–60% of BMSCs was established previously in a series of dose-response and time course studies (data not shown). BMSCs were incubated with 1.0 mM H_2_O_2_ for 6 h before the cell and nuclear morphology were assessed by standard phase contrast microscopy and Hoechst 33342 staining (Fig. 2 and 3). No differences in cell and nuclear morphology were found among all vehicle-treated control groups. In contrast, SDF-1β markedly protected Tet-Off-SDF-1β BMSCs from H_2_O_2_-induced cell death relative to Dox-suppressed (Fig. 2,3A) and Tet-Off-EV controls (Fig. 2,3B). Overall more live cells retaining the typical spindle-shaped BMSC morphology with normal round nuclei were observed compared to controls showing substantial cell loss/shrinkage and condensed nuclei, indicative of apoptosis.

**Figure 4:**
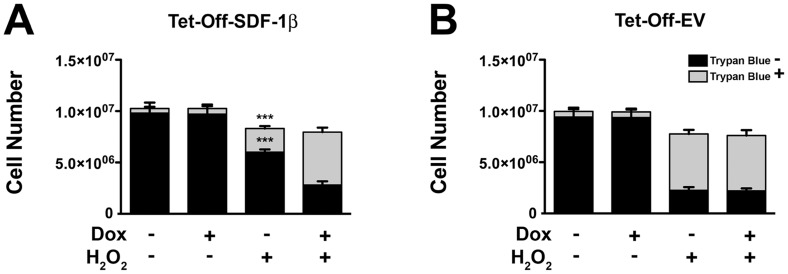
SDF-1β increases the number of surviving BMSCs following exposure to H_2_O_2_. Cell number of trypan blue-stained A) Tet-Off-SDF-1β BMSCs and B) Tet-Off-EV control BMSCs after vehicle control or H_2_O_2_ treatment. SDF-1β significantly increased the number of surviving cells (trypan blue negative) and decreased the number of dying cells (trypan blue positive) in response to H_2_O_2_ treatment compared to Dox-suppressed and Tet-Off-EV controls (6 h, ±100 ng/ml Dox, ±1.0 mM H_2_O_2_, ***p<0.0001, −Dox; H_2_O_2_ vs. +Dox; H_2_O_2_, n = 3, 3 independent experiments).

These findings led us to quantify the total number of surviving cells using a standard trypan blue staining protocol (Fig. 4). In agreement with previous results, no differences in the number of trypan blue negative and positive BMSCs were found among vehicle control groups. In contrast, SDF-1β in Tet-Off-SDF-1β BMSCs significantly increased the number of surviving cells and decreased the number of dying cells in response to H_2_O_2_ treatment compared to Dox-suppressed (Fig. 4A) and Tet-Off-EV controls (Fig. 4B) (*trypan blue negative*: Tet-Off-SDF-1β: −Dox, 6.0×10^6^±2.7×10^5^ cells, +Dox, 2.8×10^6^±3.6×10^5^ cells; Tet-Off-EV: −Dox, 2.3×10^6^±3.2×10^5^ cells, +Dox, 2.2×10^6^±2.5×10^5^ cells; p<0.0001; *trypan blue positive*: Tet-Off-SDF-1β: −Dox, 2.3×10^6^±2.5×10^5^ cells, +Dox, 5.2×10^6^±4.4×10^5^ cells; Tet-Off-EV: −Dox, 5.5×10^6^±4.1×10^5^ cells, +Dox, 5.4×10^6^±5.4×10^5^ cells; p<0.0001).

### Apoptosis and Autophagy

**Figure 5:**
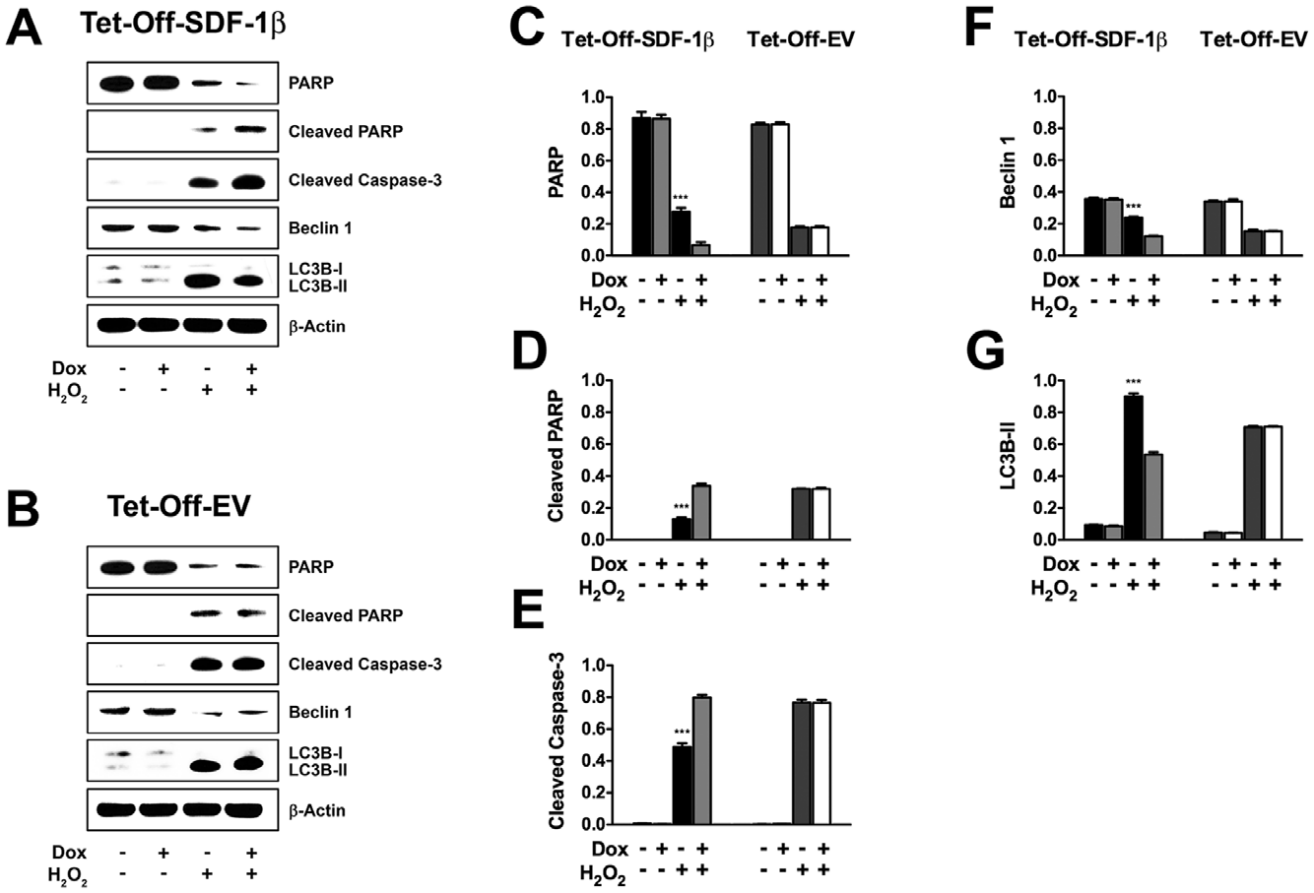
SDF-1β significantly increases key autophagy markers and decreases apoptotic markers during H_2_O_2_-induced cell death. A,B) Representative Western blots of key players involved in apoptosis and autophagy pathways. Densitometry quantification of immunoreactive bands revealed that SDF-1β significantly increased the normalized levels of C) intact PARP, decreased the levels of D) cleaved PARP and E) cleaved caspase-3, and increased the levels of F) beclin 1 and G) LC3B-II in Tet-Off-SDF-1β BMSCs in response to H_2_O_2_ treatment compared to Dox-suppressed and Tet-Off-EV controls (6 h, ±100 ng/ml Dox, ±1.0 mM H_2_O_2_, ***p<0.0001, −Dox; H_2_O_2_ vs. +Dox; H_2_O_2_, n = 3, 3 independent experiments).

To further characterize the mechanisms underlying the SDF-1β-mediated protection from cell death, we investigated key players involved in apoptosis and autophagy (Fig. 5). Western blot analysis showed that the relative levels of apoptosis markers PARP (Fig. 5C), cleaved PARP (Fig. 5D), and cleaved caspase-3 (Fig. 5E) as well as autophagy markers beclin 1 (Fig. 5F) and LC3B-II (Fig. 5G) were comparable among all vehicle control groups. In contrast, SDF-1β significantly increased the normalized levels of intact PARP, decreased the levels of cleaved PARP and cleaved caspase-3, and increased the levels of beclin 1 and LC3B-II in Tet-Off-SDF-1β BMSCs in response to H_2_O_2_ treatment (Fig. 5A–G) relative to Dox-suppressed controls (*PARP*: −Dox, 0.29±0.01, +Dox, 0.10±0.03; p<0.01; *cleaved PARP*: −Dox, 0.12±0.01, +Dox, 0.35±0.01; p<0.001; *cleaved caspase-3*: −Dox, 0.49±0.01, +Dox, 0.80±0.02; p<0.001; *beclin 1*: −Dox, 0.24±0.01, +Dox, 0.12±0.01; p<0.01; *LC3B-II*: −Dox, 0.89±0.02, +Dox, 0.53±0.02; p<0.001). No differences were found between H_2_O_2_-treated Tet-Off-EV control groups (Fig. 5B-G) (*PARP*: −Dox, 0.17±0.01, +Dox, 0.16±0.01; *cleaved PARP*: −Dox, 0.32±0.01, +Dox, 0.30±0.01; *cleaved caspase-3*: −Dox, 0.75±0.01, +Dox, 0.73±0.01; *beclin 1*: −Dox, 0.13±0.01, +Dox, 0.15±0.01; *LC3B-II*: −Dox, 0.70±0.01, +Dox, 0.71±0.01).

## Discussion

In the present study, our aim was to utilize genetically engineered Tet-Off-SDF-1β BMSCs, which conditionally overexpress SDF-1β in absence of Dox [24] as a tool to investigate whether SDF-1β could reduce H_2_O_2_-induced cell death through increasing autophagy and decreasing apoptosis in BMSCs *in vitro.* In the Tet-Off system, Dox prevents binding of the Tet-controlled transactivator to the Tet-promoter on the response vector and thus suppresses transcription of the downstream gene of interest, in our case SDF-1β [31]. We previously showed that SDF-1β mRNA expression by Tet-Off-SDF-1β BMSCs was 30-fold increased compared to controls and this increase was accompanied by a similar augmentation in SDF-1β protein levels, validating our model [24]. Importantly, we utilized both an internal control (+Dox) and a second external empty vector control, which have been shown to possess comparable levels of SDF-1 splice variant expression and downstream effects when subjected to osteogenic differentiation [24].

Previous studies have suggested a critical role of the SDF-1/CXCR4 signaling axis in maintaining proliferation and survival of stem cell populations in the BM [14]. In light of the growing interest in using BMSCs for therapeutic applications, and reports that SDF-1(α) pre-treatment or over-expression promotes the proliferation and survival of rat and human BMSCs after re-oxygenation following H_2_O_2_-treament or exposure to the apoptosis-inducing cytokine IL-4 [15], [16], we asked if SDF-1β enhances BMSCs proliferation and survival, and specifically if survival was through effects of SDF-1β on apoptotic and/or autophagic mechanisms. We showed in our system of Dox-controllable expression, that increasing SDF-1β above basal levels had no effect on BMSC proliferation over the course of 7 d compared to controls. However, we found that increasing SDF-1β significantly protected BMSCs from H_2_O_2_-induced cell death resulting in increased numbers of surviving cells relative to controls, which also retained their typical spindle-shaped morphology and normal round nuclei. Hence, our studies suggest that SDF-1β-mediated protection of BMSCs against cell death could be independent from potential effects on cell proliferation.

Upon binding to its cognate receptor CXCR4, SDF-1 has been implicated in modulating the survival-enhancing PI3-kinase/Akt and MAP-kinase/Erk1/2 signaling pathways [32]–[35], which can be blocked by the specific CXCR4 antagonist AMD3100 [15]. In addition, it was shown that SDF-1 can increase the levels of anti-apoptotic Bcl-2 and decrease the levels of pro-apoptotic Bax [15], [18]. These findings led to the investigation of the cellular mechanisms underlying the SDF-1β-dependent protection from cell death in novel Tet-Off-SDF-1β BMSCs. Therefore, we focused on key players involved in two cell death/survival processes, apoptosis and autophagy, in response to oxidative stress. Caspase activation plays a central role in the execution and completion of apoptosis. In particular, caspase-3 is critical during early apoptosis as it is involved in the proteolytic cleavage/activation of many key proteins such as PARP and other caspases [36], [37]. Therefore, we asked whether it was possible for SDF-1β to reduce caspase-3-dependent apoptosis in BMSCs. Using Western blot analysis we showed that SDF-1β over-expression significantly decreased the levels of cleaved caspase-3, resulting in decreased levels of cleaved PARP and, in turn, increased levels of intact PARP relative to controls suggesting that SDF-1β partially blocked caspase-3-dependent apoptosis in BMSCs.

It is well accepted that similar stressors can induce either apoptosis or autophagy in a context-dependent fashion. In some cases, a mixed phenotype of apoptosis and autophagy can be detected [38]. The induction of autophagy utilizes two ubiquitin-like conjugation systems as part of the vesicle elongation process. One pathway involves the covalent conjugation of Atg12 to Atg5 and the second pathway comprises the conjugation of phosphatidylethanolamine to LC3/Atg8. Lipid conjugation then leads to the conversion of the soluble form of LC3-I to the autophagosome-associated form LC3-II [39]. Another key player involved in the onset of autophagy is beclin 1. Like other BH3-only proteins, beclin 1 interacts with anti-apoptotic multi-domain proteins of the Bcl-2 family via its BH3 domain, and this interaction can be competitively disrupted to liberate beclin 1 and stimulate autophagy [40]–[46]. Despite the well-characterized signaling pathways involved, very little is known about the role of autophagy in MSC maintenance and differentiation. In a recent study it was shown that MSCs possess high levels of basal autophagy and that suppression of autophagy through knockdown of Bcl-2-xL dramatically impairs the survival and differentiation capacities of human MSCs [22]. Furthermore, the activation of autophagy has been linked to protection of MSCs from hypoxia and serum deprivation through regulating the phosphorylation of mTOR [21], [23]. In addition, a genome-wide siRNA screening revealed that under normal homeostatic conditions upregulation of autophagy requires the type III PI3-kinase, but not inhibition of mTORC1 [47]. Positive regulators of cell survival and proliferation including SDF-1/CXCR4 were identified to be involved in regulating autophagy in different cell types [47]. While these findings are intriguing and provide new insight in the role of basal autophagy in the maintenance of mammalian cells, thus far, no direct link between the survival-enhancing effects of the SDF-1/CXCR4 axis and autophagy in BMSCs has been established. Thus, we asked if the observed SDF-1β-mediated reduction in caspase-3-dependent apoptosis of BMSCs coincided with an increase in autophagy. Western blot analysis revealed that SDF-1β significantly increased the levels of LC3B-II and beclin 1 compared to controls, demonstrating an increase in autophagic markers, and suggesting that SDF-1β exerts part of its cell-protection through increasing autophagy in BMSCs. To our knowledge, this is the first report of a direct interaction of the SDF-1/CXCR4 signaling axis, and specifically the SDF-1β isoform, with autophagy in BMSCs. Future studies aim to clarify the autophagic pathways involved and to investigate the *in vivo* role of SDF-1β in protecting BMSCs from cell death upon local or systemic transplantation in models of bone disorders such as fracture and osteoporosis.

### Conclusion

In the present study we provide novel evidence that SDF-1β, a more potent splice variant compared to SDF-1α, plays a critical role in regulating BMSC survival under oxidative stress through, at least in part, increasing autophagy *in vitro*. Our studies may provide new opportunities to support BMSC-based therapies for improving regenerative medicine approaches to treat acute and chronic bone injuries.
